# Ultrasound characterization of superficial lymph nodes in HIV patients with lymphadenopathy

**DOI:** 10.3389/fmed.2025.1627659

**Published:** 2025-10-17

**Authors:** Lin Pan, Hong Liu, Huaguo Shao

**Affiliations:** ^1^Department of Ultrasound, Hangzhou Xixi Hospital Affiliated to Zhejiang Chinese Medical University, Hangzhou City, Zhejiang Province, China; ^2^Department of Pathology, Hangzhou Xixi Hospital Affiliated to Zhejiang Chinese Medical University, Hangzhou City, Zhejiang Province, China; ^3^Clinical Research Laboratory, Hangzhou Xixi Hospital Affiliated to Zhejiang Chinese Medical University, Hangzhou City, Zhejiang Province, China

**Keywords:** HIV, T cell subsets, ultrasound, lymph node, diagnosis

## Abstract

**Background and aims:**

HIV infection leads to immune deficiency and opportunistic infections, often presenting with lymphadenopathy. This study aims to characterize superficial lymph nodes in HIV patients using ultrasound and correlate these findings with T cell subset data to differentiate benign from malignant conditions.

**Methods:**

A retrospective observational study was conducted on 149 HIV patients with lymphadenopathy from March 2016 to March 2024. Ultrasound examinations were performed, and pathological results were obtained through biopsy. Statistical analysis included univariate and multivariate logistic regression to identify predictors of malignancy.

**Results:**

Ultrasound findings showed that malignant lymph nodes were larger, with a lower L/S ratio, absent hilum, and hypoechoic appearance. Immunological data revealed higher lymphocyte counts, T cell counts, and CD4^+^ T cell counts in the malignant group. The CD4^+^ T cell ratio was identified as an independent predictor of malignancy (OR 1.116, 95% CI 1.003–1.247, *p* = 0.043).

**Conclusion:**

Ultrasound combined with T cell subset analysis may assists benign and malignant lymphadenopathy in HIV patients. The CD4^+^ T cell ratio is a significant predictor of malignancy.

## Introduction

Acquired immunodeficiency syndrome (AIDS) caused by being infected with human immunodeficiency virus (HIV) causes illness and death worldwide (1, 2). HIV destroys CD4^+^ cells, leading to immune deficiency, secondary opportunistic infections and tumors in the human body. Although the incidence of opportunistic infections has significantly decreased since the advent of antiretroviral therapy (ART), they are still relatively common among HIV-infected individuals (3–5).

The lymphatic system is the main cellular immune system of the human body. Lymph is filtered by lymph nodes and flows centripetally into the human veins through lymphatic vessels to exert immune functions (6, 7). Any antigen can stimulate lymphadenopathy. Lymphadenopathy is common in HIV patients, especially superficial lymph nodes, which is the main clinical manifestation (8). CD4^+^ T cells are an important type of T cell among human immune cells and are the main targets for HIV. There is a close correlation between the occurrence of superficial lymphadenopathy and the CD4^+^ T cell count (9).

Ultrasound, as a commonly used clinical examination method, has good visualization of superficial lymph nodes, is non-radioactive, economical, and non-invasive, and can easily detect the ultrasonic characteristics of superficial enlarged lymph nodes in HIV patients (10, 11). Ultrasound-guided fine-needle aspiration biopsy is currently recognized as the best method for obtaining histopathological diagnosis without surgery. It has the advantages of wide applicability, minimal invasiveness, simple operation, and reliable examination results. The incidence of superficial lymphadenopathy is high in HIV patients, and the etiology is complex and difficult to differentiate, leading to different clinical manifestations and treatment methods. While previous studies have investigated the role of T-cell subsets in HIV infection and the ultrasonic characteristics of lymphadenopathy separately, the integration of these two types of data has been underexplored. This study aimed to retrospectively analyze the ultrasonic characteristics and T cell subset data of HIV patients with superficial lymphadenopathy, explore the differentiation of benign and malignant superficial lymphadenopathy in HIV patients.

## Methods

### Patients

This study was a retrospective observational study and included patients admitted at Hangzhou Xixi Hospital from March 2016 to March 2024. Inclusion criteria were as follows: (1) a definitive diagnosis of HIV infection; (2) enlarged lymph nodes with an unclear current diagnosis; (3) pathological results from biopsy of the enlarged lymph nodes; (4) results of T-lymphocyte subset testing. Exclusion criteria were as follows: (1) insufficient tissue obtained from lymph node biopsy, making pathological diagnosis impossible; (2) patients under 18 years of age; (3) patients with cervical lymph node lesions who had undergone anti-inflammatory treatment or chemotherapy. All HIV infections in this study were diagnosed according to the Chinese AIDS Treatment Guidelines, confirmed by the western blot method in hospital’s laboratory, and verified by the local Center for Disease Control and Prevention. This study was approved by the Clinical Research Ethics Committee of the Hangzhou Xixi Hospital.

### Ultrasound examination instruments

A GE LOGIQ E9 color Doppler ultrasound diagnostic instrument with a linear array probe (frequency range: 7.0–12.0 MHz) was used. Routine scanning of the patient’s neck, axilla, and inguinal regions was performed to observe the presence of enlarged lymph nodes and their size, shape, borders, internal echoes, lymph node hilum, and blood flow signals. Ultrasound images were captured and stored in real time. The images were analyzed jointly by a deputy chief physician in ultrasound and a physician with over 5 years of experience.

### Ultrasound-guided biopsy

The operator wore protective goggles and double-layer latex gloves and wore a fluid-resistant surgical gown for strict protection. Sterile technique was strictly followed, including routine disinfection and draping. After applying coupling gel to the probe surface, a disposable sterile probe cover was used. A disposable biopsy needle (18G-16G) produced by Bard was used. Under real-time ultrasound guidance, the disposable biopsy needle was inserted into the target lymph node to obtain tissue samples, which were then placed in a fixative solution and sent for pathological examination. The samples were also sent to the open laboratory for culture. Fungal infections of the lymph nodes were diagnosed by pathological examination and tissue culture of the biopsy specimens in our hospital’s open laboratory.

### Statistical analysis

All statistical analyses were conducted using R version 4.4.2 and a *p*-value of <0.05 was considered statistically significant. Numerical variables were shown by the medians and quantiles. Categorical variables were shown by frequencies and percentages. The non-parametric wilcoxon rank-sum test and the Chi-square test or Fisher’s exact test were performed for numerical variables and categorical variables, respectively. Considering that the amount of missing data was small and that the statistical methods used allowed for the presence of missing data, no imputation was performed on the missing data.

To analyze the impact of each variable on the outcome, we employed both univariate and multivariate logistic regression analyses. Univariate logistic regression was used to assess the impact of individual factors on the outcome variable, allowing us to identify potential predictors. Multivariate logistic regression was then applied to account for multiple factors simultaneously, adjusting for potential confounding variables and providing a more comprehensive understanding of the relationships between predictors and the outcome.

## Results

### Patient clinical data

In total, clinical and ultrasound data from 149 HIV patients with lymphadenopathy admitted at Hangzhou Xixi Hospital from March 2016 to March 2024 were collected. Based on different pathogens and pathological result, data were categorized into benign lymph node group comprising mycobacterial infection (*N* = 36), fungal infection (*N* = 32), lymphadenitis (*N* = 49), reactive lymphoid hyperplasia (*N* = 12), and malignant lymph nodes group comprising lymphoma (*N* = 13) and metastatic carcinoma (*N* = 7). Malignant cases (13.42%) were far less than benign cases. The clinical information of two patients contained missing values.

The analysis of clinical and lymphocyte subset data from HIV patients with lymphadenopathy revealed distinct patterns between benign and malignant conditions (Table 1; Supplementary Table 1). Patients with benign conditions (*N* = 129) had lower median lymphocyte counts (730 cells/μl) compared to those with malignant conditions (*N* = 20, median 1,345 cells/μl), indicating a potential immune response difference. Similarly, T cell counts were higher in the malignant group (median 1,053.5 cells/μl) than in the benign group (median 525 cells/μl), suggesting a potential T cell involvement in malignancies. The CD4^+^ T cell count was significantly higher in the malignant group (median 267 cells/μl) compared to the benign group (median 71 cells/μl), highlighting a potential role of CD4^+^ T cells in the progression or response to malignancies. The CD4^+^/CD8^+^ T cell ratio was also notably different, with a median of 0.16 in the benign group and 0.42 in the malignant group, indicating a shift in the balance of these T cell subsets. NK cell counts were higher in the malignant group (median 146 cells/μl) compared to the benign group (median 71 cells/μl), suggesting an increased presence of these cells in malignancies. B cell counts were also higher in the malignant group (median 125 cells/μl) compared to the benign group (median 55 cells/μl), indicating a potential role in the immune response to malignancies.

**Table 1:** Lymphocyte subsets in HIV patients with lymphadenopathy grouped by malignancy.

Characteristic	Overall (*N* = 149)	Benign (*N* = 129)	Malignant (*N* = 20)	*p*-value
Gender				>0.999
Female	18 (12%)	16 (12%)	2 (10%)	
Male	131 (88%)	113 (88%)	18 (90%)	
Age (years)				**0.031**
Median (Q1, Q3)	42 (32, 52)	40 (31, 51)	47.5 (38.5, 58.5)	
Min, max	20, 82	20, 77	29, 82	
HIV RNA viral loads (copies/ul)				0.335
Median (Q1, Q3)	49,300 (2,050, 304,000)	51,150 (2,250, 286,500)	5,080 (174, 439,000)	
Min, max	128, 7,160,000	128, 7,160,000	146, 528,000	
Lymphocyte count				**0.001**
Median (Q1, Q3)	770 (370, 1,390)	730 (340, 1,330)	1,345 (880, 2,065)	
Min, max	50, 5,270	50, 2,920	170, 5,270	
Lymphocyte ratio				0.178
Median (Q1, Q3)	17.4 (10, 25.2)	16.4 (9.6, 25.1)	20.15 (15.95, 25.95)	
Min, max	1.8, 80.2	1.8, 80.2	5.7, 44.5	
T cell count				**0.001**
Median (Q1, Q3)	564 (292, 1,060)	525 (251, 910)	1,053.5 (650, 1,440)	
Min, max	7.8, 3,931	7.8, 2,351	142, 3,931	
T cell ratio				0.823
Median (Q1, Q3)	77.9 (64, 83.3)	78 (63.2, 83.3)	75.85 (68.7, 82.3)	
Min, max	43.4, 95.5	43.4, 95.5	61, 91.4	
CD4^+^ T cell count				**<0.001**
Median (Q1, Q3)	85 (21, 225)	71 (17, 185)	267 (132.50, 375)	
Min, max	0, 2,461	0, 1,218	23, 2,461	
CD4^+^ T cell ratio				**<0.001**
Median (Q1, Q3)	10.6 (4.5, 22.2)	9.8 (3.5, 17.8)	21.1 (14.3, 29.1)	
Min, max	0.2, 49.7	0.2, 49.7	4.8, 46.7	
CD8^+^ T cell count				**0.007**
Median (Q1, Q3)	376 (220, 669)	361 (210, 634)	655 (333, 1,073.5)	
Min, max	17, 2,726	17, 1,852	115, 2,726	
CD8^+^ T cell ratio				0.123
Median (Q1, Q3)	57.4 (42.6, 68.5)	58.4 (43.6, 68.8)	47.2 (38, 60.05)	
Min, max	10.6, 85.1	10.6, 85.1	27.50, 81.5	
CD4^+^ T/CD8^+^ T cell ratio				**<0.001**
Median (Q1, Q3)	0.18 (0.07, 0.47)	0.16 (0.06, 0.34)	0.42 (0.26, 0.78)	
Min, max	0, 4.69	0, 4.69	0.06, 1.30	
NK cell count				**0.023**
Median (Q1, Q3)	79 (44, 184)	71 (41, 182)	146 (64, 288)	
Min, max	8, 890	8,890	27, 696	
NK cell ratio				0.523
Median (Q1, Q3)	11.7 (7.8, 18.5)	11.7 (7.8, 19.5)	11.7 (6.8, 16.5)	
Min, max	2, 46.3	2, 46.3	2.7, 29.9	
B cell count				**0.014**
Median (Q1, Q3)	59 (18, 144)	55 (18, 136)	125 (49, 230.5)	
Min, max	0, 590	1, 451	0, 590	
B cell ratio				0.773
Median (Q1, Q3)	7.9 (4.1, 13.4)	7.8 (3.90, 13.40)	9.55 (4.45, 14.1)	
Min, max	0.2, 48.5	0.2, 48.5	0.2, 30.1	

Values with *p* < 0.05 in the table are bolded to indicate statistical significance.

These findings highlight the distinct immunological profiles associated with different pathological conditions in HIV patients with lymphadenopathy. The significant differences in lymphocyte subsets suggest that these cells may play a crucial role in distinguishing between benign and malignant conditions.

### Ultrasound characteristics of swollen lymph nodes with different causes

Ultrasound findings showed that lymph nodes in the malignant group tended to be larger in size compared to those in the benign group (Table 2; Supplementary Table 2). Specifically, the long diameter (median 3.7 cm in malignant vs. 2.4 cm in benign, *p* < 0.001) and short diameter (median 2.0 cm in malignant vs. 1.1 cm in benign, *p* < 0.001) were significantly larger in malignant lymph nodes. The L/S ratio was also significantly lower in the malignant group (median 1.54 in malignant vs. 2.22 in benign, *p* < 0.001). Additionally, the presence of hilum is significantly different between the groups, with a higher frequency of absent hilum in the malignant group (82% absent in malignant vs. 46% in benign, *p* = 0.016). Echogenicity also shows a significant difference, with hypoechoic lymph nodes being more common in the malignant group (100% in malignant vs. 78% in benign, *p* = 0.044).

**Table 2:** Ultrasound characteristics in HIV patients with lymphadenopathy grouped by malignancy.

Characteristic	Overall (*N* = 149)	Benign (*N* = 129)	Malignant (*N* = 20)	*p*-value
Long diameter (cm)				**<0.001**
Median (Q1, Q3)	2.5 (2, 3.25)	2.4 (2, 3.1)	3.7 (2.6, 6.8)	
Min, max	1.2, 12	1.2, 4.6	1.5, 12	
Short diameter (cm)				**<0.001**
Median (Q1, Q3)	1.15 (0.9, 1.5)	1.1 (0.9, 1.5)	2 (1.2, 4.6)	
Min, max	0.4, 15	0.4, 2.9	0.9, 15	
L/S ratio				**<0.001**
Median (Q1, Q3)	2.17 (1.72, 2.65)	2.22 (1.79, 2.75)	1.54 (1.38, 2.1)	
Min, max	0.8, 5.6	1.04, 5.6	0.8, 4.22	
Shape				0.094
Irregular	43 (30%)	35 (27%)	8 (47%)	
Regular	102 (70%)	93 (73%)	9 (53%)	
Echogenicity				**0.044**
Hyperechoic	28 (19%)	28 (22%)	0 (0%)	
Hypoechoic	117 (81%)	100 (78%)	17 (100%)	
Border				0.596
Defined	137 (94%)	120 (94%)	17 (100%)	
Undefined	8 (5.5%)	8 (6.3%)	0 (0%)	
Calcification	1 (0.7%)	1 (0.8%)	0 (0%)	>0.999
Hilum				**0.016**
Absent	73 (50%)	59 (46%)	14 (82%)	
Present	52 (36%)	50 (39%)	2 (12%)	
Thinner	20 (14%)	19 (15%)	1 (5.9%)	
Cystic degeneration	27 (19%)	25 (20%)	2 (12%)	0.740
Edema of peripheral soft tissue	40 (28%)	38 (30%)	2 (12%)	0.155
Blood flow signal				0.125
Abundant	77 (53%)	64 (50%)	13 (76%)	
No	15 (10%)	14 (11%)	1 (5.9%)	
Poor	53 (37%)	50 (39%)	3 (18%)	
Puncture site				**0.026**
Abdominal lymph nodes	3 (2.0%)	1 (0.8%)	2 (10%)	
Left axillary lymph nodes	14 (9.4%)	12 (9.3%)	2 (10%)	
Left cervical lymph nodes	67 (45%)	61 (47%)	6 (30%)	
Left inguinal lymph nodes	5 (3.4%)	3 (2.3%)	2 (10%)	
Right axillary lymph nodes	8 (5.4%)	8 (6.2%)	0 (0%)	
Right cervical lymph nodes	42 (28%)	37 (29%)	5 (25%)	
Right inguinal lymph nodes	10 (6.7%)	7 (5.4%)	3 (15%)	

Values with *p* < 0.05 in the table are bolded to indicate statistical significance.

**Figure 1:**
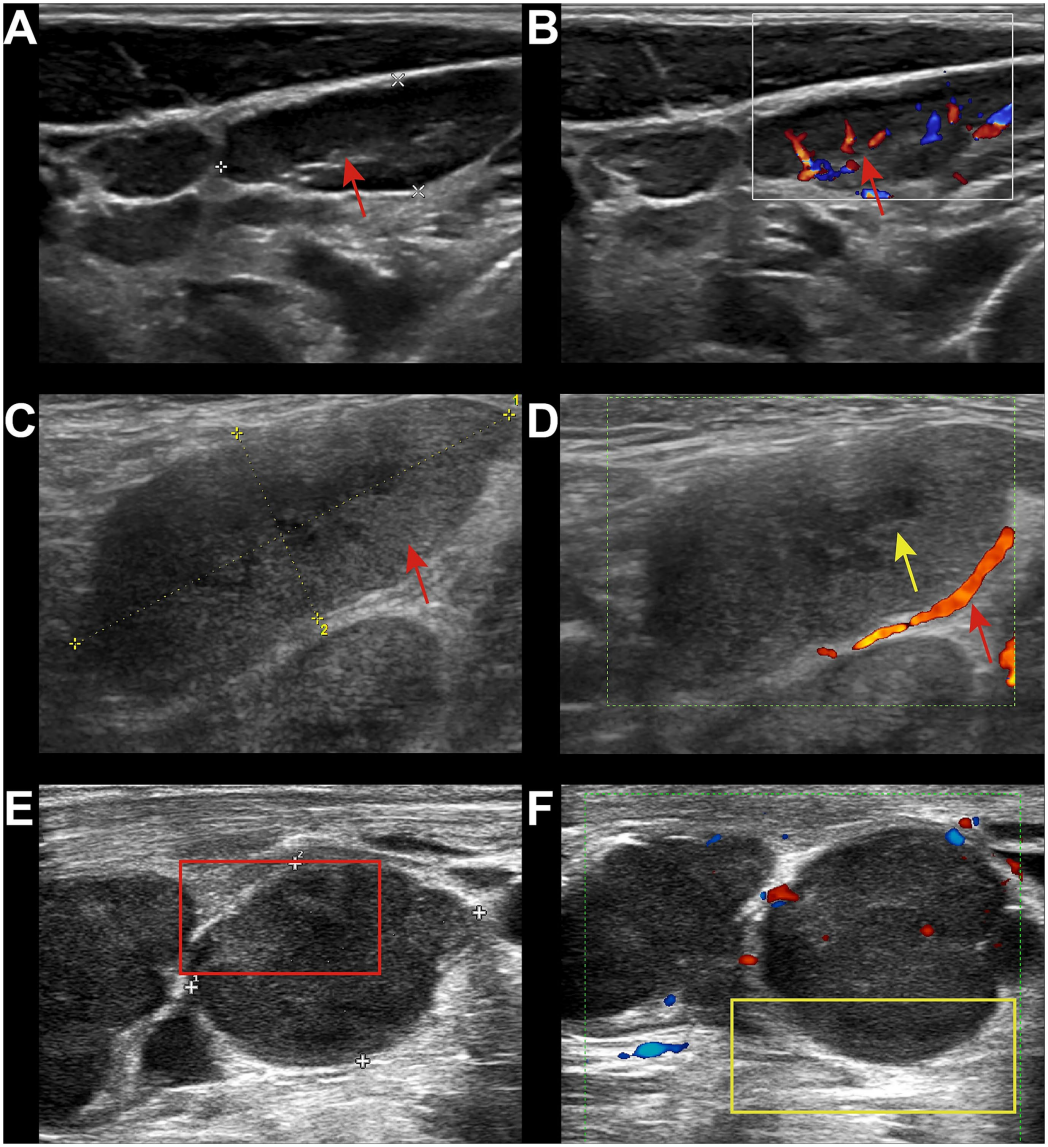
Ultrasound images of benign group. **(A)** 24-year-old male, diagnosed with HIV infection for 4 years. Left cervical lymph node showed enlargement with regular shape, clear margins, and visible lymph node hilum (red arrow). **(B)** CDFI showed abundant hilar blood flow (red arrow). The pathological diagnosis was reactive lymphoid hyperplasia. **(C)** 36-year-old male, diagnosed with HIV infection for 6 years. Multiple lymph node enlargements in the right axilla with regular shape had increased internal echogenicity (red arrow). **(D)** A few cystic changes were visible (yellow arrow), with the absence of the lymph node hilum. CDFI showed minimal peripheral blood flow (red arrow). The pathological diagnosis was fungal infection, with the final culture result identifying Talaromyces marneffei infection. **(E)** 48-year-old male, diagnosed with HIV infection for 8 months. Multiple lymph node enlargements were in the right neck with regular shape and heterogeneous internal echogenicity (red box). **(F)** The lymph node hilum was absent, and surrounding soft tissue edema was present (yellow box). CDFI showed minimal peripheral blood flow signals. The pathological diagnosis was TB.

**Figure 2:**
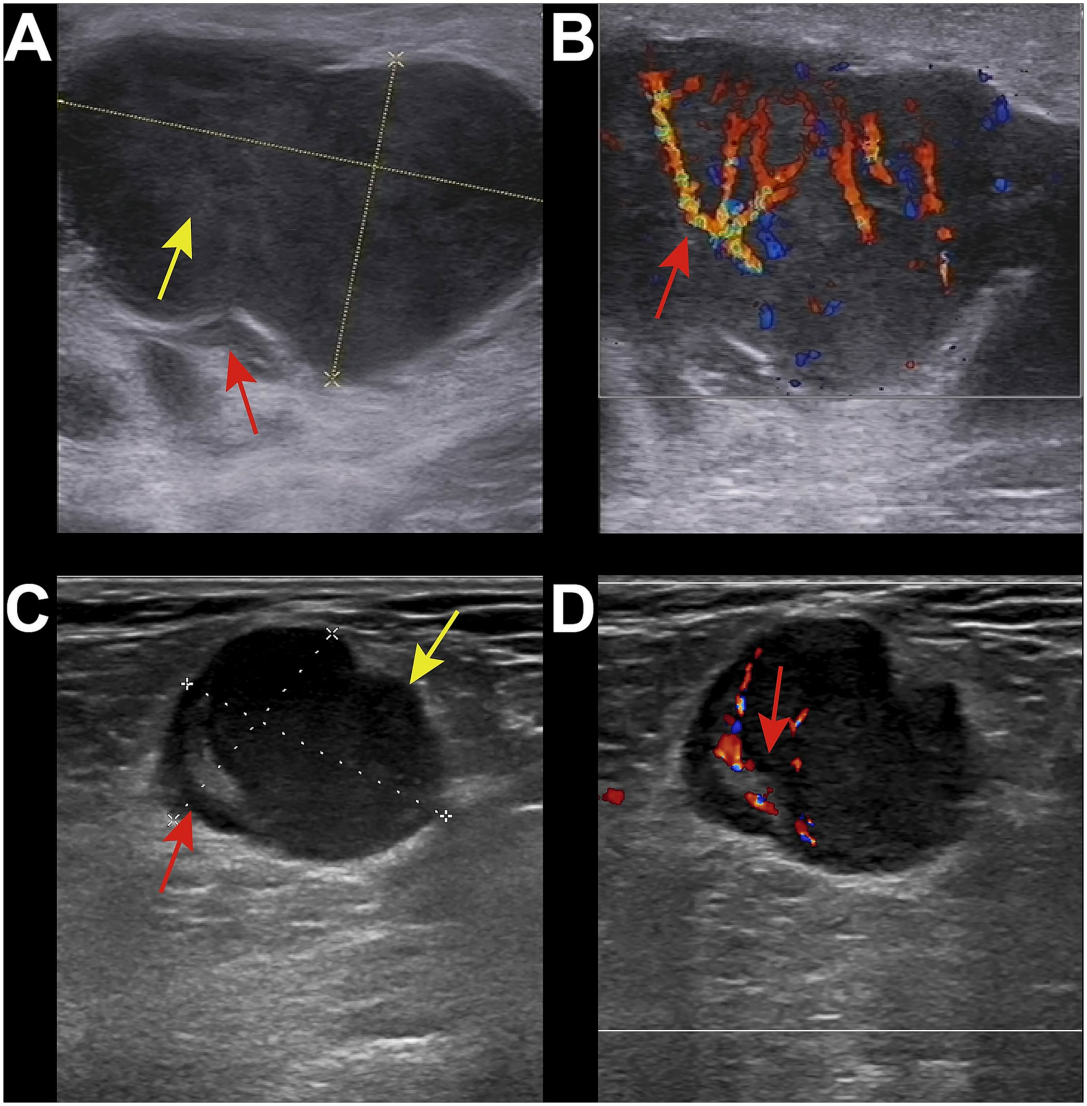
Ultrasound images of malignant group. **(A)** 34-year-old male, diagnosed with HIV infection for 1 year. Right inguinal lymph node enlargement was with local capsule indentation and irregular shape (red arrow). The internal echogenicity showed a reticular pattern (yellow arrow), with the absence of the lymph node hilum. **(B)** CDFI showed abundant blood flow signals (red arrow). The pathological diagnosis was non-Hodgkin lymphoma-diffuse large B-cell lymphoma. **(C)** 50-year-old male, diagnosed with HIV infection for 5 years. Enlarged lymph node was in the right inguinal region with irregular shape and significant asymmetric cortical thickening (red arrow). The lymph node hilum was present but compressed and eccentric (green arrow). The internal echogenicity was relatively uniform. **(D)** CDFI showed abundant hilar blood flow signals (red arrow). The pathological diagnosis was metastatic squamous cell carcinoma.

Reactive lymphadenopathy was commonly characterized by regular lymph node shape, clear margins, and the presence of a lymph node hilum, with abundant blood flow signals demonstrated on Color Doppler Flow Imaging (CDFI) (Figures 1A,B). Tuberculous lymphadenopathy varied in presentation depending on the disease stage. In the early stage, it resembled reactive lymphadenopathy. In the middle and late stages, lymph nodes fused with each other, resulting in irregular shapes and ulceration, forming sinus tracts (Figures 1C,D). Cystic changes were relatively common within the lymph nodes, and internal calcifications and surrounding soft tissue edema may be observed. Fungal infections typically presented with regular lymph node shape and mostly uniform increased echogenicity within the lymph nodes (Figures 1E,F). A few cases may showed cystic changes. The lymph node hilum was often absent or thinned. Blood flow patterns on CDFI were variable, with no significant edema in the surrounding tissues and no calcifications observed. Lymphoma involvement of lymph nodes usually manifested as round or oval lymph nodes with a full appearance, which fused (Figures 2A,B). When fused, the lymph nodes were often large, sometimes exceeding 10 cm, and the shape was usually irregular. The lymph node hilum was frequently absent. With low gain settings, the lymph node may appear anechoic, while increasing the gain reveals a reticular echogenic pattern within the lymph node. Metastatic lymph nodes were often multiple, with round or irregular shapes and an L/S ratio <2 (Figures 2C,D). The margins were clear, but may appeared blurred if the lymph node capsule was invaded. The lymph node hilum may be thinned, compressed eccentrically, or even completely absent. The cortex was irregularly thickened, and the internal echogenicity was heterogeneous.

### Potential predictors for malignancy

The results of the univariate and multivariate logistic regression analyses for predicting lymph node malignancy were presented in Table 3. Only statistically significant results are shown in the table due to too many results of calculations.

**Table 3:** Univariate and multivariate logistic regression analysis for malignancy of lymph nodes.

Characteristic	Reference	Univariate analysis		Multivariate analysis	
		OR (95% CI)	*p*-value	OR (95% CI)	*p*-value
Age (years)	–	1.035 (1.001–1.07)	0.041		
Lymphocyte count	–	1.001 (1–1.001)	0.002		
T cell count	–	1.001 (1–1.002)	0.001		
CD4^+^ T cell count	–	1.002 (1.001–1.004)	0.010		
CD4^+^ T cell ratio	–	1.058 (1.02–1.099)	0.003	1.116 (1.003–1.247)	0.043
B cell count	–	1.004 (1.001–1.008)	0.009		
CD8^+^ T cell count	–	1.001 (1–1.002)	0.005		
Long diameter (cm)	–	2.329 (1.553–3.949)	<0.001		
Short diameter (cm)	–	3.829 (2.037–8.38)	<0.001		
L/S ratio	–	0.31 (0.124–0.674)	0.006		
Shape	Irregular	0.357 (0.129–0.983)	0.044		
Echogenicity	Hyperechoic	0.123 (0.007–0.63)	0.045		
Hilum	Absent	0.143 (0.022–0.538)	0.012		

In the univariate analysis, several factors were significantly associated with lymph node malignancy. Age (OR 1.035, 95% CI 1.001–1.07, *p* = 0.041), lymphocyte count (OR 1.001, 95% CI 1–1.001, *p* = 0.002), T cell count (OR 1.001, 95% CI 1–1.002, *p* = 0.001), CD4^+^ T cell count (OR 1.002, 95% CI 1.001–1.004, *p* = 0.010), CD4^+^ T cell ratio (OR 1.058, 95% CI 1.02–1.099, *p* = 0.003), B cell count (OR 1.004, 95% CI 1.001–1.008, *p* = 0.009), CD8^+^ T cell count (OR 1.001, 95% CI 1–1.002, *p* = 0.005), long diameter (OR 2.329, 95% CI 1.553–3.949, *p* < 0.001) and short diameter (OR 3.829, 95% CI 2.037–8.38, *p* < 0.001) were identified as significant predictors for malignancy.

The L/S ratio (OR 0.31, 95% CI 0.124–0.674, *p* = 0.006), irregular shape (OR 0.357, 95% CI 0.129–0.983, *p* = 0.044), hyperechoic echogenicity (OR 0.123, 95% CI 0.007–0.63, *p* = 0.045), and absence of hilum (OR 0.143, 95% CI 0.022–0.538, *p* = 0.012) were also significant predictors but inversely associated with malignancy.

In the multivariate analysis, only the CD4^+^ T cell ratio remained significant (OR 1.116, 95% CI 1.003–1.247, *p* = 0.043). This suggested that among the evaluated factors, the CD4^+^ T cell ratio was probably an independent predictor of lymph node malignancy.

## Discussion

The lymphatic system, as one of the most important immune systems, consists of lymph nodes and associated lymphatic vessels. When pathological changes occur, patients often present with symptoms of enlarged superficial lymph nodes, which are commonly found in the neck, axilla, and inguinal regions. These areas are the easiest for patients to examine themselves. Among them, enlargement of the cervical lymph nodes is the most common. Patients usually visit the doctor due to observing or feeling a lump in the neck. In all HIV patients, about 35% present with lymphadenopathy as the initial symptom (12).

Lymph node reactive hyperplasia refers to the reactive proliferation of lymphocytes and histiocytes in the lymph nodes caused by various injuries and stimuli, leading to lymph node enlargement. It is the most common cause of lymphadenopathy. However, its proportion in HIV-infected individuals is lower than that in HIV-negative populations, which is consistent with the research results of Sun et al. (13). Various cellular reactions can be encountered in the lymph nodes, which are manifestations of immune activation. These reactions alter the normal histology and main functions of the lymph nodes, and any part of the lymph nodes may be affected (14). On ultrasound, such lymph nodes often appear oval-shaped, with a clear and intact capsule. The lymph node volume is enlarged, and the ratio of the long axis to the short axis is usually greater than two. The lymph node hilum is often present, and typical hilar blood flow is shown on CDFI. The fatty hilum sign is observed on ultrasound, which is characterized by an enlarged lymph node hilum, an increased central echogenic medulla, and a thinned cortex. This change is more likely to occur in the axillary and inguinal regions.

In our study, ultrasound examination revealed that the volume of lymph nodes in the malignant group was larger than that in the benign group. The long and short diameters of the lymph nodes in the malignant group were significantly greater than those in the benign group, and the L/S ratio in the malignant group was also significantly lower. These findings are consistent with those of ÖZTÜRK et al., who reported that the short-axis size of benign lymph nodes ranged from 5 to 28 mm, while that of malignant lymph nodes ranged from 5 to 53 mm. The L/S ratios for benign and malignant lymph nodes were 0.23 to 1 and 0.33 to 1, respectively (15). Sun et al. conducted a study on 204 cases of lymphadenopathy and found significant differences in the transverse size, hilar echogenicity, long-to-transverse diameter ratio, echo characteristics, and color Doppler flow patterns among benign lymph nodes, metastatic lymph nodes, and lymphomas (16). In numerous studies, an L/S ratio of ≥2 has been considered indicative of benign lymph nodes, while metastatic lymph nodes have typically been described as round with an L/S ratio of <2 (17). In the malignant group, the incidence of missing lymph node hilum was higher, and hypoechoic lymph nodes were more prevalent. These observations align with the findings of Fischerova et al., who demonstrated that partial infiltration of lymph nodes by malignancy often presents as eccentric cortical thickening. This is characterized by uneven cortical thickening that displaces the residual hyperechoic lymph node hilum and medulla to the periphery of the lymph node. Compared with adjacent soft tissue, the cortex appears to have heterogeneous or markedly hypoechoic echogenicity (14).

Fungal infections are the most common opportunistic infections in AIDS patients in the late stage and are also one of the main causes of death (18). Lymphokines released by T lymphocytes accelerate epidermal keratinization and dandruff formation. The shedding of dandruff helps to avoid fungal infections. Immune damage caused by type IV hypersensitivity mediated by T lymphocytes control and kill fungi, thereby terminating the infection (19, 20). Humoral immunity also plays a protective role against fungal infections, but this is only effective when the antibodies produced by the body are in a good immune state. Since the immune systems of AIDS patients are all damaged to varying degrees, they are more susceptible to fungal infections. In our study, the most common fungal infection was caused by Mucor, followed by Cryptococcus. Some cases could not be identified as specific fungal species, and the final diagnosis was limited to fungal infection, which may be related to the laboratory culture methods. On ultrasound, lymph nodes infected with fungi often appear round or oval-shaped, with a continuous and intact capsule. The internal echoes are usually uneven, and the parenchymal echoes are diffusely increased. Calcification is rarely seen.

At present, tuberculosis (TB) remains an important infectious disease threatening human health and is also a common opportunistic infection in HIV-infected individuals. About 50% of HIV-infected patients suffer from extrapulmonary TB, among whom about 35% have concurrent lymph node TB (21). In our study, mycobacterial infections were mainly caused by TB, while non-tuberculous mycobacterial infections were mainly caused by the *Mycobacterium avium*-intracellulare complex, which may be related to the climate and environment of our city. In patients with normal immune function, infection with MTB lead to the formation of typical granulomas composed of fused epithelioid cells in the affected tissues and organs to resist the spread of the pathogenic pathogen. However, in patients with compromised immune function, the number of multinucleated giant cells in the TB foci will be significantly reduced after infection. They lack an immune response to the pathogenic pathogen and are more likely to form atypical granulomas with loosely arranged epithelioid cells (22). The ultrasound manifestations of TB lymph nodes are diverse. In the early stage, the shape can be regular, and the lymph node hilum may be present or absent. In the middle and late stages, as the necrotic components in the lymph nodes increase and the pressure rises, the lymph nodes may rupture, resulting in an irregular shape. Tuberculous abscesses can also be seen around them. Compression may produce a sense of movement, and even cause the formation of non-healing sinus tracts that penetrate the skin. Coarse calcification is also relatively common in the middle and late stages of tuberculous lymph nodes (23). No significant ultrasound features were observed in lymph nodes infected with *Mycobacterium avium* complex in our study, which may be related to the small sample size of our group. After reviewing the literature, no one has been found to summarize the corresponding ultrasound characteristics for it.

Malignant lesions in HIV-infected individuals are mainly lymphomas and metastatic cancers. The incidence of lymphoma is significantly higher than that in the general population, especially the risk of non-Hodgkin’s lymphoma, which is 60 to 200 times higher than that in the general population (24, 25). This increased risk is due to the immunosuppression caused by HIV, which promotes virus-induced carcinogenesis (26). Fine-needle aspiration cytology (FNAC) is an important diagnostic method for lymphoma because it distinguishes lymphoma from cancer (13). Cytological examination of FNAC is an important morphological and immunohistochemical study of lymphoma cells and cell blocks, which has been reported to be highly accurate in the diagnosis of lymphoma and further classify subtypes in many cases (27). On ultrasound, lymph nodes involved by lymphoma often have a regular shape and lower echoes than the surrounding muscle tissue. Some lymphomas in the inguinal or axillary regions grow very large, even exceeding 10 cm. At this time, the shape may become irregular, and the internal echoes are chaotic. Clinically, patients visit the doctor due to the discovery of a large mass. CDFI shows rich blood flow signals, mainly peripheral or mixed types. Metastatic cancer is mainly squamous cell carcinoma, with only one case of metastatic papillary thyroid carcinoma, which may be related to the small sample size. When metastatic cancer lymph nodes are small, they often have a regular shape. When the lesions enlarge, they also merge to form irregular masses. Currently, the manifestations vary according to the type of primary tumor. They appear in patches, have corresponding distribution characteristics, and some may have calcification. In lymph nodes metastasized from papillary thyroid carcinoma, typical punctate calcifications can be seen, and some may have high-echo areas inside. This is due to the accumulation of thyroglobulin produced by the metastatic foci. Some may undergo cystic changes, and when they are completely cystic, it is difficult to distinguish them from other cysts.

In results, we identified the CD4^+^ T cell ratio as an independent predictor of malignancy in HIV-associated lymphadenopathy. The CD4^+^ T cell ratio provides a normalized measure of the CD4^+^ T cell population relative to the overall lymphocyte pool (28). This normalization is crucial because absolute CD4^+^ T cell counts can be influenced by various factors, including overall lymphocyte proliferation, inflammation, and the stage of HIV infection. By normalizing the CD4^+^ T cell count, the ratio provides a more context-specific measure of immune function, which is less affected by these confounding factors. CD4^+^ T cells are critical for orchestrating the immune response against tumors. They help activate CD8^+^ cytotoxic T cells, which directly kill tumor cells, and they support the function of other immune cells, such as macrophages and dendritic cells (29). The CD4^+^ T cell ratio provides a more comprehensive view of the immune system’s ability to respond to and control tumor growth (30). In HIV-infected individuals, where the immune system is compromised, maintaining a higher CD4^+^ T cell ratio is particularly important for effective immune surveillance and tumor control (31). While absolute CD4^+^ T cell counts are important for assessing overall immune function, they do not provide the same level of normalization and contextualization as the CD4^+^ T cell ratio. Absolute counts can be influenced by various factors, including overall lymphocyte proliferation and the stage of HIV infection, which can introduce variability and reduce the predictive power of the model. The CD4^+^ T cell ratio, by normalizing these counts, provides a more stable and reliable measure of immune function, particularly in the context of tumor immunity.

This study has many limitations. Firstly, the number of malignant samples was relatively small, which may have limited the statistical power of our analysis and the generalizability of our findings. A larger sample size, particularly with more positive cases, would provide a more robust basis for drawing conclusions and could enhance the reliability of our results. Secondly, this was a single-center study, which means that our findings may not be representative of the broader population. Thirdly, we did not keep detailed records of the individual judgments made by the observers regarding the ultrasonic characteristics. This lack of documentation prevents us from conducting a thorough analysis of inter-observer variability and the specific criteria used for assessing the ultrasound images. Future studies should implement a more rigorous recording system to capture these details, which would facilitate a deeper understanding of the diagnostic process and improve the accuracy of the results. Additionally, we did not perform imputation for missing data. While the amount of missing data was small and the statistical methods used allowed for its presence, this decision may have introduced some bias into our analysis. In future studies, we will consider appropriate imputation techniques to address missing data, which can help to reduce potential biases and improve the completeness and accuracy of the dataset.

## Conclusion

In conclusion, this study highlights the importance of ultrasound in differentiating benign and malignant superficial lymphadenopathy in HIV patients. Ultrasound characteristics, such as lymph node size, shape, echogenicity, and the presence of hilum, combined with T cell subset data, provide valuable diagnostic information. The CD4^+^ T cell ratio was identified as an independent predictor of malignancy. These findings suggest that ultrasound, along with immunological markers, can assist in the clinical management of HIV patients with lymphadenopathy.

## Data Availability

The original contributions presented in the study are included in the article/Supplementary material, further inquiries can be directed to the corresponding author.
