# Supplementary material for: Ultrasound characterization of superficial lymph nodes in HIV patients with lymphadenopathy
Source: Front Med (Lausanne). 2025 Oct 17;12:1627659. doi: 10.3389/fmed.2025.1627659 (PMC12575233; doi:10.3389/fmed.2025.1627659)
Supplement: Supplementary file 1 [file Table_1.docx]

Supplementary Table 1. Lymphocyte subsets in HIV patients with lymphadenectasis grouped among different groups.

| Characteristic | Overall  (N = 149) | Mycobacterial infection  (N = 36) | Fungal infection  (N = 32) | Lymphadenitis  (N = 49) | Reactive lymphoid hyperplasia  (N = 12) | Lymphoma  (N = 13) | Metastatic carcinoma  (N = 7) | *P*-value |
| --- | --- | --- | --- | --- | --- | --- | --- | --- |
| Gender |  |  |  |  |  |  |  | 0.798 |
| female | 18 (12%) | 7 (19%) | 3 (9.4%) | 5 (10%) | 1 (8.3%) | 1 (7.7%) | 1 (14%) |  |
| male | 131 (88%) | 29 (81%) | 29 (91%) | 44 (90%) | 11 (92%) | 12 (92%) | 6 (86%) |  |
| Age (years) |  |  |  |  |  |  |  | 0.134 |
| median  (Q1,Q3) | 42.00(32.00,48.00) | 36.00(30.00,53.00) | 43.00(31.00,51.00) | 43.50(34.50,56.00) | 43.00(34.00,58.00) | 50.00(45.00,70.00) | 42.00(32.00,48.00) |  |
| min,max | 23.00,73.00 | 23.00,64.00 | 20.00,77.00 | 28.00,74.00 | 29.00,80.00 | 45.00,82.00 | 23.00,73.00 |  |
| HIV RNA viral loads (copies/ul) |  |  |  |  |  |  |  | 0.503 |
| median  (Q1,Q3) | 49,300.00(2,050.00,304,000.00) | 52,200.00(1,600.00,304,000.00) | 66,850.00(1,300.00,261,500.00) | 43,900.00(3,200.00,247,000.00) | 108,600.00(33,750.00,848,500.00) | 221,245.00(1,832.00,483,500.00) | 3,408.00(146.00,6,670.00) |  |
| min,max | 128.00,7,160,000.00 | 179.00,1,370,000.00 | 200.00,1,540,000.00 | 128.00,3,200,000.00 | 2,610.00,7,160,000.00 | 174.00,528,000.00 | 146.00,6,670.00 |  |
| Lymphocyte count |  |  |  |  |  |  |  | **<0.001** |
| median  (Q1,Q3) | 770.00(370.00,1,390.00) | 590.00(350.00,850.00) | 365.00(250.00,745.00) | 995.00(585.00,1,495.00) | 1,400.00(580.00,1,930.00) | 1,460.00(690.00,1,920.00) | 1,300.00(1,050.00,2,210.00) |  |
| min,max | 50.00,5,270.00 | 50.00,2,430.00 | 100.00,2,660.00 | 170.00,2,830.00 | 260.00,2,920.00 | 170.00,5,270.00 | 840.00,2,330.00 |  |
| Lymphocyte ratio |  |  |  |  |  |  |  | **0.003** |
| median  (Q1,Q3) | 17.40(10.00,25.20) | 13.70(6.20,22.10) | 12.25(8.35,18.80) | 18.55(14.60,31.20) | 24.35(17.95,34.55) | 20.20(15.00,24.10) | 20.10(17.00,29.50) |  |
| min,max | 1.80,80.20 | 2.80,46.80 | 2.70,46.80 | 1.80,80.20 | 6.20,45.90 | 5.70,38.40 | 7.60,44.50 |  |
| T cell count |  |  |  |  |  |  |  | **<0.001** |
| median  (Q1,Q3) | 564.00(292.00,1,060.00) | 453.00(204.00,703.00) | 257.50(185.50,515.50) | 702.50(464.50,1,190.50) | 898.00(378.50,1,445.00) | 1,120.00(542.00,1,505.00) | 823.00(659.00,1,375.00) |  |
| min,max | 7.80,3,931.00 | 24.00,2,214.00 | 7.80,1,878.00 | 114.00,2,191.00 | 185.00,2,351.00 | 142.00,3,931.00 | 641.00,2,067.00 |  |
| T cell ratio |  |  |  |  |  |  |  | 0.583 |
| median  (Q1,Q3) | 77.90(64.00,83.30) | 78.40(68.30,82.90) | 78.35(70.40,83.25) | 77.95(62.60,83.75) | 68.10(56.75,80.10) | 78.40(71.50,83.50) | 64.00(62.20,76.30) |  |
| min,max | 43.40,95.50 | 43.40,95.50 | 43.70,94.80 | 48.20,95.00 | 53.40,94.10 | 68.70,91.40 | 61.00,88.70 |  |
| CD4^+^T cell count |  |  |  |  |  |  |  | **<0.001** |
| median  (Q1,Q3) | 85.00(21.00,225.00) | 59.00(10.00,135.00) | 14.00(4.00,41.50) | 152.50(57.50,218.00) | 132.50(97.00,557.00) | 156.00(112.00,366.00) | 334.00(260.00,384.00) |  |
| min,max | 0.00,2,461.00 | 0.00,654.00 | 1.00,432.00 | 1.00,984.00 | 18.00,1,218.00 | 23.00,2,461.00 | 260.00,517.00 |  |
| CD4^+^T cell ratio |  |  |  |  |  |  |  | **<0.001** |
| median  (Q1,Q3) | 10.60(4.50,22.20) | 9.30(4.20,16.30) | 3.45(1.25,8.05) | 13.40(5.60,23.70) | 20.70(9.45,31.50) | 16.50(12.40,22.60) | 24.80(20.00,30.90) |  |
| min,max | 0.20,49.70 | 0.20,44.30 | 0.30,32.50 | 0.30,49.70 | 5.40,41.70 | 4.80,46.70 | 15.80,32.60 |  |
| CD8^+^T cell count |  |  |  |  |  |  |  | **<0.001** |
| median  (Q1,Q3) | 376.00(220.00,669.00) | 336.00(184.00,527.00) | 224.00(162.50,408.50) | 508.00(282.00,836.50) | 533.00(296.50,896.50) | 720.00(335.00,1,179.00) | 554.00(323.00,968.00) |  |
| min,max | 17.00,2,726.00 | 17.00,1,852.00 | 58.00,1,749.00 | 100.00,1,365.00 | 63.00,1,566.00 | 115.00,2,726.00 | 297.00,1,452.00 |  |
| CD8^+^T cell ratio |  |  |  |  |  |  |  | **0.008** |
| median  (Q1,Q3) | 57.40(42.60,68.50) | 60.60(43.60,69.50) | 66.45(56.30,71.80) | 54.45(39.80,68.20) | 44.75(35.25,59.10) | 53.10(45.60,67.90) | 42.60(34.70,46.50) |  |
| min,max | 10.60,85.10 | 29.00,83.30 | 31.70,84.40 | 10.60,82.70 | 18.30,85.10 | 35.90,81.50 | 27.50,62.30 |  |
| CD4^+^T/CD8^+^T cell ratio |  |  |  |  |  |  |  | **<0.001** |
| median  (Q1,Q3) | 0.18(0.07,0.47) | 0.16(0.08,0.32) | 0.05(0.02,0.12) | 0.23(0.10,0.52) | 0.46(0.15,0.90) | 0.29(0.20,0.49) | 0.59(0.36,0.85) |  |
| min,max | 0.00,4.69 | 0.00,1.34 | 0.01,0.79 | 0.01,4.69 | 0.09,2.00 | 0.06,1.30 | 0.36,1.12 |  |
| NK cell count |  |  |  |  |  |  |  | **<0.001** |
| Median  (Q1,Q3) | 79.00(44.00,184.00) | 71.00(44.00,162.00) | 42.50(24.50,136.50) | 97.50(57.00,200.00) | 224.50(63.50,290.00) | 159.00(60.00,236.00) | 133.00(66.00,343.00) |  |
| min,max | 8.00,890.00 | 10.00,282.00 | 8.00,646.00 | 15.00,890.00 | 37.00,570.00 | 27.00,696.00 | 62.00,376.00 |  |
| NK cell ratio |  |  |  |  |  |  |  | 0.503 |
| median  (Q1,Q3) | 11.70(7.80,18.50) | 11.70(9.30,21.40) | 13.10(7.80,16.50) | 10.55(7.30,16.30) | 18.65(10.25,27.20) | 13.20(7.00,16.00) | 8.60(5.90,26.40) |  |
| min,max | 2.00,46.30 | 3.80,46.30 | 4.20,40.10 | 2.70,36.10 | 2.00,37.80 | 2.70,27.30 | 5.70,29.90 |  |
| B cell count |  |  |  |  |  |  |  | **<0.001** |
| median  (Q1,Q3) | 59.00(18.00,144.00) | 35.00(11.00,79.00) | 25.00(11.50,65.50) | 87.00(38.50,149.50) | 153.00(20.50,243.00) | 123.00(23.00,202.00) | 127.00(107.00,316.00) |  |
| min,max | 0.00,590.00 | 1.00,412.00 | 3.00,305.00 | 3.00,451.00 | 4.00,364.00 | 0.00,590.00 | 69.00,433.00 |  |
| B cell ratio |  |  |  |  |  |  |  | 0.317 |
| median  (Q1,Q3) | 7.90(4.10,13.40) | 6.60(3.80,11.00) | 7.45(2.90,13.10) | 9.50(5.10,14.65) | 7.25(3.00,16.25) | 6.90(3.40,11.20) | 14.80(6.40,19.60) |  |
| min,max | 0.20,48.50 | 0.20,29.60 | 0.90,48.50 | 0.80,39.30 | 1.00,21.20 | 0.20,19.30 | 5.10,30.10 |  |
| Values with *P* < 0.05 in the table are bolded to indicate statistical significance. | | | | | | | | |
