# Supplementary material for: Ultrasound characterization of superficial lymph nodes in HIV patients with lymphadenopathy
Source: Front Med (Lausanne). 2025 Oct 17;12:1627659. doi: 10.3389/fmed.2025.1627659 (PMC12575233; doi:10.3389/fmed.2025.1627659)
Supplement: Supplementary file 2 [file Table_2.docx]

Supplementary Table 2. Ultrasound characteristics in HIV patients with lymphadenectasis grouped among different groups.

| Characteristic | Overall  (N = 149) | Mycobacterial infection  (N = 36) | Fungal infection  (N = 32) | Lymphadenitis  (N = 49) | Reactive lymphoid hyperplasia  (N = 12) | Lymphoma  (N = 13) | Metastatic carcinoma  (N = 7) | *P*-value |
| --- | --- | --- | --- | --- | --- | --- | --- | --- |
| Long diameter (mm) |  |  |  |  |  |  |  | **<0.001** |
| median  (Q1,Q3) | 2.50(2.00,3.25) | 2.90(2.15,3.50) | 2.40(2.00,3.15) | 2.20(2.00,2.80) | 2.20(1.90,2.65) | 3.90(2.70,7.85) | 2.80(1.70,4.30) |  |
| min,max | 1.20,12.00 | 1.20,4.50 | 1.20,4.60 | 1.60,3.70 | 1.20,3.60 | 2.20,12.00 | 1.50,6.90 |  |
| Short diameter (mm) |  |  |  |  |  |  |  | **<0.001** |
| median  (Q1,Q3) | 1.15(0.90,1.50) | 1.40(1.15,1.95) | 1.10(0.90,1.55) | 1.00(0.80,1.20) | 0.80(0.70,1.00) | 2.75(1.35,6.15) | 1.60(1.00,2.90) |  |
| min,max | 0.40,15.00 | 0.60,2.90 | 0.60,2.60 | 0.40,1.80 | 0.50,1.50 | 0.90,15.00 | 1.00,4.60 |  |
| L/S ratio |  |  |  |  |  |  |  | **<0.001** |
| median  (Q1,Q3) | 2.17(1.72,2.65) | 2.00(1.56,2.46) | 2.05(1.69,2.57) | 2.43(2.00,2.86) | 2.58(2.29,2.88) | 1.55(1.27,2.22) | 1.50(1.48,2.08) |  |
| min,max | 0.80,5.60 | 1.04,3.11 | 1.09,3.89 | 1.27,5.60 | 2.18,4.00 | 0.80,4.22 | 1.40,2.31 |  |
| Shape |  |  |  |  |  |  |  | **0.001** |
| irregular | 43 (30%) | 18 (51%) | 3 (9.4%) | 12 (24%) | 2 (17%) | 5 (50%) | 3 (43%) |  |
| regular | 102 (70%) | 17 (49%) | 29 (91%) | 37 (76%) | 10 (83%) | 5 (50%) | 4 (57%) |  |
| Echogenicity |  |  |  |  |  |  |  | **<0.001** |
| hyperechoic | 28 (19%) | 4 (11%) | 20 (63%) | 3 (6.1%) | 1 (8.3%) | 0 (0%) | 0 (0%) |  |
| hypoechoic | 117 (81%) | 31 (89%) | 12 (38%) | 46 (94%) | 11 (92%) | 10 (100%) | 7 (100%) |  |
| Border |  |  |  |  |  |  |  | **0.011** |
| defined | 137 (94%) | 28 (80%) | 32 (100%) | 48 (98%) | 12 (100%) | 10 (100%) | 7 (100%) |  |
| undefined | 8 (5.5%) | 7 (20%) | 0 (0%) | 1 (2.0%) | 0 (0%) | 0 (0%) | 0 (0%) |  |
| Calcification | 1 (0.7%) | 1 (2.9%) | 0 (0%) | 0 (0%) | 0 (0%) | 0 (0%) | 0 (0%) | 0.662 |
| Hilum |  |  |  |  |  |  |  | **-** |
| absent | 73 (50%) | 29 (83%) | 13 (41%) | 14 (29%) | 3 (25%) | 8 (80%) | 6 (86%) |  |
| present | 52 (36%) | 3 (8.6%) | 8 (25%) | 31 (63%) | 8 (67%) | 1 (10%) | 1 (14%) |  |
| thinner | 20 (14%) | 3 (8.6%) | 11 (34%) | 4 (8.2%) | 1 (8.3%) | 1 (10%) | 0 (0%) |  |
| Cystic degeneration | 27 (19%) | 11 (31%) | 6 (19%) | 8 (16%) | 0 (0%) | 0 (0%) | 2 (29%) | 0.083 |
| Edema of peripheral soft tissue | 40 (28%) | 24 (69%) | 3 (9.4%) | 11 (22%) | 0 (0%) | 2 (20%) | 0 (0%) | **<0.001** |
| Blood flow signal |  |  |  |  |  |  |  | - |
| abundant | 77 (53%) | 18 (51%) | 15 (47%) | 24 (49%) | 7 (58%) | 8 (80%) | 5 (71%) |  |
| no | 15 (10%) | 7 (20%) | 4 (13%) | 3 (6.1%) | 0 (0%) | 0 (0%) | 1 (14%) |  |
| poor | 53 (37%) | 10 (29%) | 13 (41%) | 22 (45%) | 5 (42%) | 2 (20%) | 1 (14%) |  |
| Puncture site |  |  |  |  |  |  |  | - |
| abdominal lymph nodes | 3 (2.0%) | 0 (0%) | 1 (3.1%) | 0 (0%) | 0 (0%) | 2 (15%) | 0 (0%) |  |
| left axillary lymph nodes | 14 (9.4%) | 0 (0%) | 3 (9.4%) | 7 (14%) | 2 (17%) | 2 (15%) | 0 (0%) |  |
| left cervical lymph nodes | 67 (45%) | 19 (53%) | 17 (53%) | 20 (41%) | 5 (42%) | 5 (38%) | 1 (14%) |  |
| left inguinal lymph nodes | 5 (3.4%) | 0 (0%) | 0 (0%) | 2 (4.1%) | 1 (8.3%) | 0 (0%) | 2 (29%) |  |
| right axillary lymph nodes | 8 (5.4%) | 1 (2.8%) | 0 (0%) | 6 (12%) | 1 (8.3%) | 0 (0%) | 0 (0%) |  |
| right cervical lymph nodes | 42 (28%) | 14 (39%) | 10 (31%) | 10 (20%) | 3 (25%) | 3 (23%) | 2 (29%) |  |
| right inguinal lymph nodes | 10 (6.7%) | 2 (5.6%) | 1 (3.1%) | 4 (8.2%) | 0 (0%) | 1 (7.7%) | 2 (29%) |  |
| Values with *P* < 0.05 in the table are bolded to indicate statistical significance. | | | | | | | | |
